# Data-driven adaptive hybrid models for exchange rate return forecasting

**DOI:** 10.3389/fdata.2026.1917723

**Published:** 2026-09-11

**Authors:** Olumide Sunday Adesina, Lawrence Ogechukwu Obokoh

**Affiliations:** Johannesburg Business School, University of Johannesburg, Johannesburg, South Africa

**Keywords:** currency exchange, forecasting, hybrid models, machine learning, time-series

## Abstract

**Background:**

Exchange-rate return forecasting is challenging because financial time series may exhibit linearity, nonlinearity, regime-switching behavior, and volatility. To address these complexities, two adaptive hybrid forecasting frameworks were developed: ATW-HyF A, which dynamically combines ARIMA, SETAR, and ANN forecasts using inverse-variance weighting, and ATW-HyF B, which extends the framework by incorporating a GARCH(1,1) volatility layer to model the conditional variance of forecast errors.

**Methods:**

The robustness of the proposed frameworks was assessed using simulated data and three foreign exchange return series: USD/NGN, EUR/USD, and GBP/USD. Forecast performance was evaluated within a rolling-origin forecasting framework and compared with competing forecasting models.

**Results:**

The simulation results showed that the ATW-HyF models achieved the lowest forecast errors among the competing models. However, their performance on the empirical exchange-rate series was mixed, with other models achieving lower forecast errors for some currency pairs. In particular, the ARIMA-ANN hybrid performed best for USD/NGN and EUR/USD, whereas ATW-HyF B performed best for GBP/USD.

**Discussion:**

The findings indicate that forecasting performance depends on the characteristics of the underlying exchange-rate series rather than on model complexity alone. Combining models with complementary predictive strengths can improve forecasting performance, particularly when the data exhibit linear, nonlinear, and volatility-related patterns. The incorporation of adaptive weighting and volatility modeling therefore provides a flexible approach to exchange-rate return forecasting under evolving market conditions.

## 1 Introduction

Exchange return forecasting remains challenging in financial econometrics because currency markets exhibit inherent volatility and nonlinearity, which leads to weak predictability. Prediction accuracy is a priority in exchange rate prediction because it provides essential information for policymakers, investors, and multinational firms, who rely on such predictions to make informed decisions regarding international trade, monetary policy, and risk management (Alexandridis et al., 2024). The empirical literature for over three decades maintains its conflicting results on exchange rate predictability and forecasting model performance (Cheung et al., 2005; Rossi, 2013). Meese and Rogoff (1983) established the foundation for contemporary research on exchange rate predictability by demonstrating that structural exchange rate models fail to exceed the forecasting accuracy of a random walk model. The result shows remarkable strength because it remains consistent through different periods and various datasets, which researchers commonly refer to as the “exchange rate disconnect puzzle.” Further studies confirmed this initial finding and demonstrated that macroeconomic fundamentals only deliver limited forecasting capabilities at short-term intervals (Engel and West, 2005; Moosa, 2013). The latest research continues to demonstrate that exchange rate relationships experience instability, thereby creating challenges in developing reliable forecasting methods (Beckmann and Czudaj, 2017; Byrne et al., 2016).

Various studies have adopted modeling techniques that integrate microstructure components with nonlinear dynamic systems because these modeling techniques are an improvement on the existing models because financial series are hardly linear (Adesina and Obokoh, 2024; Evans and Lyons, 2005). The authors developed micro-based exchange rate models that utilize order flow information to present a new approach for determining exchange rate values. The new developments demonstrate that international currency markets operate through complex mechanisms that require advanced research methods because conventional models lack the ability to describe these dynamics. The traditional linear time series models, which include autoregressive integrated moving average (ARIMA), provide easy modeling understanding and deliver accurate results for short-term predictions, but often fail if there are complexities in the data.

Although machine learning and hybrid models have demonstrated improved predictive performance in many financial applications, their superiority is not universal (Khashei and Bijari, 2011; Solomon et al., 2026; Zhang, 2003). Their effectiveness often depends on data characteristics, forecast horizon, and model specification, suggesting that increased model complexity alone does not guarantee better exchange-rate forecasts (El Kadri et al., 2025; Makridakis et al., 2018).

The analysis of currency exchange requires volatility modeling as an essential component, which was first introduced by Engle (1982) and Bollerslev (1986). The authors developed Autoregressive Conditional Heteroscedasticity and Generalized ARCH models to effectively model financial time series behavior, which includes volatility clustering and changing risk levels. While GARCH-type models remain the benchmark for modeling conditional volatility, empirical evidence suggests that improvements in volatility forecasts do not necessarily translate into more accurate mean forecasts, particularly for exchange-rate returns characterized by weak serial dependence (Huang and Luo, 2024). Volatility information serves a vital function in risk management while its correct application helps to enhance forecasting systems. Volatility information serves a vital function in risk management while its correct application helps to enhance forecasting systems.

The adaptive weighting mechanism adopted in this study is grounded in the theory of optimal forecast combination introduced by Bates and Granger (1969), which demonstrates that combining multiple forecasts can reduce forecast uncertainty relative to individual models. Under the minimum-variance principle, forecasts with lower error variance provide more precise information and should therefore receive greater weights in the combined forecast. Since the precision of forecasting models changes over time in exchange-rate markets because of regime shifts and volatility clustering, fixed combination weights may become suboptimal. Consequently, this study updates inverse-variance weights recursively using rolling forecasting performance, allowing the contribution of each component model to evolve according to its recent predictive reliability while preserving the theoretical advantages of minimum-variance forecast combination. However, many existing combination methods employ fixed or equal weights, implicitly assuming that model performance remains stable over time, an assumption that is rarely satisfied in foreign exchange markets (Timmermann, 2006; Wang et al., 2022).

The development of hybrid financial models seeks to combine the essential strengths of different types of methods. The combination can either be two or three models, depending on the nature or complexity of the data. For example, the hybrid of three models may include linear, nonlinear, and machine learning methods. Hybrid models apply their forecasting capabilities to multiple financial data, such as stock prices, price volatility, and currency exchange rates (Kristjanpoller and Minutolo, 2018; Özdoğan et al., 2026; Adesina and Obokoh, 2026). Although many studies report that hybrid models outperform standalone methods, the reported gains vary considerably across datasets and market conditions. This inconsistency suggests that forecasting performance depends not only on model architecture but also on how effectively complementary information is integrated (Adesina and Obokoh, 2025).

Despite decades of research, there is still no consensus on whether increasingly sophisticated forecasting models consistently outperform simpler alternatives. While some studies favor nonlinear and hybrid approaches, others argue that forecasting gains are often sample-specific and diminish under changing market conditions (Kim and Kim, 2020; Rossi, 2013).

The current study introduces an adaptive forecasting framework that unifies linear, nonlinear, and volatility models. It introduces time-varying weights (ATW-HyF) which captures various complexities that exist in foreign exchange data. Consequently, highlighting various properties of time-series that exhibit nonlinear, regime switching, and volatility clustering, exhibiting how the properties of time-series data differ from one another, which also affects model performance.

### 1.1 Dynamic forecasting

Though dynamic forecast combination methods exist in forecasting research, many hybrid forecasting systems in exchange-rate prediction rely on fixed or static weighting structures. The models do not adopt evolving nonlinear market regimes, structural breaks, and volatility interactions (Timmermann, 2006; Wang et al., 2022).

Recent forecasting studies have been increasing interested in adaptive, hybrid learning setups, with the blend of several streams of information to model nonlinear patterns in financial time series. Gao et al. (2025) proposed a multi-scale adaptive stock fusion framework, to track the temporal movements across different horizons, while Keun Yun (2025) showed how crucial it is to bring in exogenous market sentiment data when building predictive systems. Ma and Yuan (2024) worked on a regularized ensemble forecasting framework that basically stitches together multiple predictors to improve robustness, and Su et al. (2024) proposed an adaptive spatial–temporal learning layout for handling dynamic market relationships. Also, Zhang et al. (2022) used attention-based mechanisms to strengthen the way temporal dependencies are represented in financial forecasting.

Collectively, these studies demonstrate that adaptive and ensemble learning methods enhance financial forecasting by exploiting multiple sources of predictive information. However, most focus on improving individual model architectures or incorporating additional explanatory variables rather than addressing how model contributions should evolve as market conditions change. Consequently, the challenge remains to develop forecasting frameworks that dynamically reallocate model weights according to changing predictive reliability.

Limited attention has been paid to forecasting frameworks that dynamically update forecast weights while simultaneously integrating linear, nonlinear, regime-switching, and volatility-sensitive components within a unified architecture (Bates and Granger, 1969; Stock and Watson, 2004). No single forecasting model can consistently explain the complex and evolving behavior of exchange rates, as predictive performance often changes across market conditions. To address this challenge, the proposed framework adopts inverse-variance weighting, which assigns greater importance to models with lower forecast uncertainty while reducing the influence of less reliable models. By updating these weights over time, the framework adapts to changing market dynamics and volatility, leading to more stable and robust forecasts. This approach is consistent with recent studies demonstrating that adaptive forecast combination methods improve exchange rate forecasting by effectively integrating the strengths of multiple models (Alexandridis et al., 2024; Huang and Luo, 2024). The outcome of this study will provide answers to the question as to whether there could be a single model that that fit all time financial time series data irrespective of the statistics properties of the data.

### 1.2 Contribution

This study proposes an Adaptive Time-Varying Weighted Hybrid Forecasting (ATW–HyF) framework that dynamically combines ARIMA, SETAR, and ANN forecasts using inverse-RMSE weights updated recursively over a rolling forecasting window, rather than relying on conventional fixed-weight forecast combinations. In addition, the proposed ATW–HyF B extends the adaptive framework by incorporating a GARCH(1,1) volatility layer to model time-varying forecast uncertainty and volatility clustering, providing a unified mean–variance forecasting framework with inverse-variance weighting for exchange-rate returns.

The proposed framework combines ARIMA, SETAR, and ANN because these models capture complementary characteristics of exchange-rate returns, namely linear dependence, regime-switching behavior, and nonlinear relationships. Although recent deep learning architectures such as Long Short-Term Memory (LSTM) networks and Transformers have demonstrated promising forecasting performance, their superiority is not universal. Their performance often depends on the availability of very large datasets, extensive hyperparameter optimisation, and substantial computational resources. Furthermore, several comparative studies have shown that classical statistical and hybrid models remain competitive for noisy financial return series exhibiting weak serial dependence and rapidly changing market conditions. Therefore, rather than proposing another deep learning architecture, this study focuses on developing an adaptive forecast combination strategy that dynamically integrates complementary forecasting models according to their recent predictive performance. This allows the contribution of individual models to evolve over time while maintaining computational efficiency and model interpretability. The proposed framework should therefore be viewed as an adaptive benchmark against which more computationally intensive deep learning approaches can be evaluated in future studies.

This study does not claim that ARIMA–SETAR–ANN is universally superior to LSTM or Transformer-based models. Instead, it investigates whether adaptive forecast weighting can improve predictive performance by exploiting the complementary strengths of classical statistical and nonlinear forecasting models under evolving market conditions. The study introduces an adaptive framework that recursively updates inverse-variance weights within an ARIMA–SETAR–ANN forecasting architecture and integrates a GARCH layer for conditional volatility. The framework is comprehensively evaluated against standalone models, conventional hybrid models, and benchmark forecasts using rolling-origin forecasting and Harvey–Leybourne–Newbold predictive accuracy tests.

The research findings demonstrate new insights showing how model complexity affects predictive accuracy, which will guide researchers, practitioners, and policymakers in various ways.

## 2 Methodology

### 2.1 Research framework

This study proposes an Adaptive Time-Varying Hybrid Forecasting (ATW–HyF) framework for one-step-ahead exchange-rate return forecasting. The framework combines three complementary forecasting paradigms. The linear statistical modeling (ARIMA), nonlinear regime-switching modeling (SETAR), and machine-learning forecasting (Artificial Neural Network, ANN) within an expanding rolling-origin forecasting environment. Different from the conventional forecast combination methods that employ fixed or equal weights, the proposed framework updates the contribution of each individual forecast recursively according to its recent forecasting performance.

The framework consists of two complementary components.

ATW–HyF A produces adaptive point forecasts by dynamically combining the one-step-ahead forecasts generated by ARIMA, SETAR, and ANN.

ATW–HyF B extends ATW–HyF A by modeling the conditional variance of the hybrid forecast residuals using a GARCH(1,1) process, thereby simultaneously forecasting the conditional mean and conditional volatility.

The complete framework consists of six sequential stages:

(i) Transform currency exchange returns into continuously compounded returns.
(ii) Generate recursive one-step-ahead forecasts using an expanding rolling-origin procedure.
(iii) Estimate ARIMA, SETAR and ANN models at each forecast origin.
(iv) Dynamically combine the forecasts using adaptive inverse rolling-RMSE weights.
(v) Estimate conditional volatility from the hybrid residuals using GARCH(1,1).
(vi) Evaluate forecasting performance using RMSE, MAE, MAPE sMAPE, MASE, and the Harvey–Leybourne–Newbold (HLN)-corrected Diebold–Mariano test.

### 2.2 Data preprocessing

Let *P*_*t*_ denote the observed exchange rate at time *t*.

To stabilize the variance and remove non-stationarity, continuously compounded returns are computed in Equation 1 as


$$\begin{array}{l} r_{t}=ln\left(\frac{P_{t}}{P_{t-1}}\right) \end{array}$$ (1)

Where *r*_*t*_ is the log returns.

### 2.3 Rolling-origin forecasting framework

Forecast evaluation is conducted using an expanding rolling-origin (walk-forward) procedure.

Suppose the initial estimation window contains *W*_*o*_ observations.

At forecast origin *t*, the information set is


$$\begin{array}{l} F_{t}=\left\{r_{1},r_{2},\ldots ,r_{t}\right\} \end{array}$$ (2)

Each forecasting model is estimated using only $F_{t}$ in Equation 2, thereby avoiding look-ahead bias.

Let


$$\begin{array}{l} f_{j}\;\left(\cdot \right),\;j=1,\;\ldots ,J \end{array}$$

represent forecasting model *j*.

The corresponding one-step-ahead forecast is provided in Equation 3


$$\begin{array}{l} {\hat{r}}_{j,t+1}=f_{j}\;\left(F_{1}\right) \end{array}$$ (3)

In this study, *J* = 3, corresponding to ARIMA, ANN, and SETAR.

After the realized observation *r*_1 + 1_ becomes available, the estimation window expands to


$$\begin{array}{l} {\;F}_{t+1}=F_{t}\cup \left\{{\;r}_{t+1}\right\} \end{array}$$

the procedure is repeated until the end of the sample.

### 2.4 Individual forecasting models

The adaptive framework combines three complementary forecasting models.

#### 2.4.1 ARIMA

The *ARIMA*(1, 0, 1) model captures the linear temporal dependence in exchange-rate returns and is expressed in Equation 4 as


$$\begin{array}{l} r_{t}=\text{ϕ}r_{t-1}+\text{θ}\varepsilon_{t-1}+\varepsilon_{t}, \end{array}$$ (4)

where, ϕ is the autoregressive coefficient, θ is the moving-average coefficient, and, $\varepsilon_{t}\sim N\left(0,\sigma^{2}\right)$.

The corresponding one-step-ahead forecast is provided in Equation 5


$$\begin{array}{l} {\hat{r}}_{A,\;t+1}=\text{ϕ}r_{t}+\text{θ}{\hat{\varepsilon }}_{t} \end{array}$$ (5)

#### 2.4.2 SETAR

The nonlinear component is represented by a two-regime SETAR model in Equation 6,


$$r_{t}=\{\begin{array}{c} \alpha_{1}+\sum_{i=1}^{p_{1}}\phi_{1i}r_{t-i}+\varepsilon_{t},\;r_{t-d}\leq \tau \;\\ \alpha_{2}+\sum_{i=1}^{p_{2}}\phi_{2i}r_{t-i}+\varepsilon_{t},\;r_{t-d}>\tau , \end{array}$$ (6)

where

- τ denotes the threshold parameter;
- *d* is the delay parameter;
- *p*_1_ and *p*_2_ denote the autoregressive orders within each regime.

The resulting forecasting is found in Equation 7


$${\hat{r}}_{S,\;t+1}=\{\begin{array}{c} \alpha_{1}+\sum_{i=1}^{p_{1}}\phi_{1i}r_{t+1-i},\;r_{t+1-d}\leq \tau \;\\ \alpha_{2}+\sum_{i=1}^{p_{2}}\phi_{2i}r_{t+1-i},\;r_{t+1-d}>\tau , \end{array}$$ (7)

#### 2.4.3 Artificial neural network

The Artificial Neural Network (ANN) approximates nonlinear functional relationships between past and future exchange-rate returns. Let the input vector at forecast origin *t* be


$$\begin{array}{l} {\text{x}}_{t}={\left(r_{t},\;r_{t-1},\;r_{t-2},\;r_{t-p+1}\right)}^{T} \end{array}$$ (8)

From Equation 8, *p* denotes the number of lagged returns used as network inputs.

The output of hidden neuron *k* is denoted by h_*k*_.

For a feedforward neural network containing one hidden layer, the hidden neuron activations are computed as


$$\begin{array}{l} h_{k}=g\left(\overset{p}{\underset{i=1}{\sum }}w_{ik}x_{i}+b_{k}\right) \end{array}$$

Where, *w*_*ik*_ is the weight connecting input *i* to hidden neuron *k*, *b*_*k*_ is the hidden-layer bias term, and *g*(·) is a nonlinear activation function consistent with the computational implementation, the Rectified Linear Unit (ReLU) activation function is adopted,


$$\begin{array}{l} g\left(z\right)=\max\left(0,z\right) \end{array}$$

Is the Rectified Linear Unit (ReLU) activation function.

The one-step ahead ANN forecast is presented in Equation 9 as


$$\begin{array}{l} {\hat{r}}_{N,\;t+1}=c+\overset{H}{\underset{k=1}{\sum }}w_{k}h_{k,} \end{array}$$ (9)

where *H* is the number of hidden neurons, *w*_*k*_ is the weight connecting hidden neuron *k* to the output neuron, and *c* is the output-layer bias.

### 2.5 Model hybridization

The study uses three types of hybridization. The ARIMA–ANN, ARIMA–SETAR, SETAR–ANN uses equal-weight ensemble combination. As follows:


$$\begin{array}{l} {\hat{r}}_{AA,t}=\frac{1}{2}\left({\hat{r}}_{ARIMA,t}+{\hat{r}}_{ANN,t}\right)\\ {\hat{r}}_{AS,t}=\frac{1}{2}\left({\hat{r}}_{ARIMA,t}+{\hat{r}}_{SETAR,t}\right)\;\\ {\hat{r}}_{SA,t}=\frac{1}{2}\left({\hat{r}}_{SETAR,t}+{\hat{r}}_{ANN,t}\right)\;\\ {\hat{r}}_{ASA,t}=\frac{1}{2}\left({\hat{r}}_{ARIMA,t}+{\hat{r}}_{SETAR,t}+{\hat{r}}_{ANN,t}\right) \end{array}$$

The novelty of the study lies in ATW-HyF A and ATW-HyF B. Instead of fixed weights (1/2 or 1/3), they use adaptive performance-based weights, discussed in Sections 2.6 and 2.7, respectively.

### 2.6 Adaptive time-varying hybrid forecasting (ATW–HyF A)

The proposed hybrid framework dynamically combines the forecasts generated by ARIMA, SETAR, and ANN. Unlike conventional forecast combinations employing fixed weights, the proposed framework estimates adaptive weights recursively according to recent forecasting performance.

#### 2.6.1 Forecast error

After the realized return becomes available, the forecast error of model *j* is provided in Equation 10


$$\begin{array}{l} e_{j,t}=r_{t}-{\hat{r}}_{j,t}, \end{array}$$ (10)

#### 2.6.2 Rolling forecast performance

Forecast performance is measured using the rolling Root Mean Square Error (RMSE) as provided in Equation 11,


$$\begin{array}{l} RMSE_{J,t}=\sqrt{\frac{1}{m}\overset{t}{\underset{i=t-m+1}{\sum }}e_{j,i}^{2}} \end{array}$$ (11)

where,

*m* is rolling window length (number of observations in the moving window),

*e*_*j, i*_ is forecast error of model *j* at time *i*,

*t* is the current forecast origin.

#### 2.6.3 Performance score

Each forecast first receives a performance score, the score is provided in Equation 12,


$$\begin{array}{l} S_{j,t}=\frac{1}{RMSE_{j,t}+\varepsilon } \end{array}$$ (12)

where


$$\begin{array}{l} \varepsilon =10^{-8} \end{array}$$

Introducing ε is to prevent numerical instability. Lower RMSE therefore produces larger performance scores.

#### 2.6.4 Adaptive weight estimation

The adaptive weight associated with forecast *j* in Equation 13


$$\begin{array}{l} w_{j,t}=\frac{S_{j,t}}{\sum_{k=1}^{3}S_{k,t}}, \end{array}$$ (13)

Subject to


$$\begin{array}{l} w_{j,t}\geq 0, \end{array}$$

and


$$\begin{array}{l} \overset{3}{\underset{k=1}{\sum }}w_{j,t}=1 \end{array}$$ (14)

The weights in Equations 13 and 14 are estimated from the recent forecasting performance of the individual models but are applied to the corresponding one-step-ahead forecasts.

#### 2.6.5 Proposed hybrid forecast equation

The proposed adaptive hybrid forecast is


$$\begin{array}{l} {\hat{r}}_{t+1}^{ATW-HyF_{A}}={w_{A,t}\hat{r}}_{A,\;t+1}+{w_{S,t}\hat{r}}_{S,\;t+1}+{w_{N,t}\hat{r}}_{N,\;t+1} \end{array}$$ (15)

Equation 15 defines the proposed ATW–HyF A framework as a convex combination of the ARIMA, SETAR and ANN forecasts whose weights evolve over time according to recent predictive performance.

### 2.7 Volatility-augmented hybrid forecasting (ATW–HyF B)

Although Equation 15 provides an adaptive point forecast, financial returns exhibit volatility clustering that cannot be represented adequately by the conditional mean alone. Thereby producing adaptive conditional mean forecasts while modeling the conditional variance of the hybrid forecast residuals. Thus, ATW-HyF B extends ATW-HyF A by providing both point forecasts and forecasts of time-varying uncertainty.

The hybrid forecast residual is provided in Equation 16


$$\begin{array}{l} {e_{t}=r}_{t}-{\hat{r}}_{t}^{ATW-HyF_{A}} \end{array}$$ (16)

The residual process is assumed to satisfy the process in Equation 17,


$$\begin{array}{l} e_{t}=\sigma_{t}z_{t},\;{\;z}_{t}\sim N\left(0,1\right) \end{array}$$ (17)

With conditional variance in Equation 18


$$\begin{array}{l} \sigma^{2}=\omega +\alpha e_{t-1}^{2}+\beta {\sigma^{2}}_{t-1}, \end{array}$$ (18)

where

ω is the long-run variance component;

α measures the short-run impact of recent forecast shocks;

β captures volatility persistence.

The parameter space satisfies: ω>0, α≥0, β≥0, and α+β < 1

The one-step-ahead volatility forecast is provided in Equation 19


$$\begin{array}{l} {\hat{\sigma }}_{t+1}^{2}=\omega +\alpha {ê}_{t}^{2}+\beta {\sigma^{2}}_{t} \end{array}$$ (19)

Consequently, the complete framework is given in Equation 20


$$\begin{array}{l} ATW-HyF_{B}=\left({\hat{r}}_{t+1}^{ATW-HyF_{A}},\;{\hat{\sigma }}_{t+1}^{2}\right), \end{array}$$ (20)

which jointly forecasts the conditional mean and conditional variance of exchange-rate returns.

The fixed hybrid of weighted ARIMA, SETAR, and ANN uses equal weight is presented in Equation 21


$$\begin{array}{l} {\hat{r}}_{t}^{FHW}=\frac{1}{3}\left({\hat{r}}_{t}^{ARIMA}+{\hat{r}}_{t}^{SETAR}+{\hat{r}}_{t}^{ANN}\right) \end{array}$$ (21)

### 2.8 Simulation

The time series *y*_*t*_ is generated using a two-regime threshold autoregressive (TAR) process. The process is provided in Equation 22


$$y_{t}=\{\begin{array}{c} 0.5y_{t-1}+\varepsilon_{t},\text{ if }y_{t-1}<0\;\\ -0.3y_{t-1}+\varepsilon_{t},\text{ if }y_{t-1}\geq 0 \end{array}$$ (22)

Which was preceded by generating a sequence of independent and identically distributed (i.i.d.) random errors ε_*t*_~*N*(0, 1), *t* = 1, 2, …, *n*.

The simulated data were generated from a nonlinear threshold autoregressive process with two regimes. Specifically, the data generating process is defined as *y*_*t*_ = 0.5*y*_*t*−1_+ε_*t*_ when *y*_*t*−1_ < 0, and *y*_*t*_ = −0.3*y*_*t*−1_+ε_*t*_ otherwise, where $\varepsilon_{t}N\sim \left(0,1\right)$. The simulated sample was 600 with initial value *y*_0_ = 0, and a fixed random seed as 123 to ensure reproducibility.

The study employs a rolling-origin (walk-forward) validation framework in which models are iteratively trained on an expanding window of historical observations and evaluated using one-step-ahead forecasts. This approach ensures that all predictions are strictly out-of-sample and avoids look-ahead bias, providing a robust assessment of forecasting performance. Although the simulated process does not explicitly contain conditional heteroskedasticity, the volatility layer may still capture local variability generated by regime-switching dynamics.

### 2.9 Data description

The study uses daily exchange rate data of three important currency pairs, USD/ Nigerian Naira (NGN) for the emerging market pair, and Major international currency pairs, EUR/USD and GBP/USD. Where USD is the United States Dollar, EURO is the European Euro, and GBP is the Great Britain Pound. The data span the period from April 4, 2016, to April 6, 2026, with 2,610 observations for each series obtained from https://www.investing.com/. The data include open, high, low, and closing prices. The closing prices were used in this study. Daily frequency data enables researchers to study exchange rate movements that occur within short to medium time periods while collecting sufficient data points to create accurate hybrid forecasting models.

### 2.10 Forecast evaluation

The models are evaluated with the metrics provided in Equations 23–28, and32

Root Mean Square Error


$$\begin{array}{l} RMSE=\sqrt{\frac{1}{N}{\sum \left(r_{t}-\hat{r_{t}}\right)}^{2}} \end{array}$$ (23)

Mean Absolute Error


$$\begin{array}{l} MAE=\frac{1}{N}\sum |r_{t}-\hat{r_{t}}| \end{array}$$ (24)

where *r*_*t*_ is the true value and ${\hat{r}}_{t}\;$is the predicted value, *N* is the total number of data points.

Mean Absolute Error


$$\begin{array}{l} MAPE=\frac{1}{N}\sum |\frac{r_{t}-{\hat{r}}_{t}\;}{r_{t}}|\times 100 \end{array}$$ (25)

Symmetric Mean Absolute Percentage Error


$$\begin{array}{l} sMAPE=\frac{100}{N}\overset{N}{\underset{t=1}{\sum }}\frac{\left|r_{t}-{\hat{r}}_{t}\right|}{\frac{\left(\left|r_{t}|-|{\hat{r}}_{t}\right|\right)}{2}} \end{array}$$ (26)

Mean Absolute Scaled Error


$$\begin{array}{l} MASE=\frac{MAE_{i}}{MAE_{j}} \end{array}$$ (27)

Where *MAE*_*i*_ is the MAE for the new model and the *MAE*_*j*_is the MAE for the original model.

If *MASE* < 1, then the new model performs better, otherwise the model perform better.

Theil's Inequality Coefficient (Theil's U)


$$\begin{array}{l} U=\frac{\sqrt{\frac{1}{n}\sum_{t=1}^{N}{\sum \left(r_{t}-\hat{r_{t}}\right)}^{2}}}{\sqrt{\frac{1}{N}\sum_{t=1}^{N}{\sum r_{t}}^{2}}+\sqrt{\frac{1}{N}\sum_{t=1}^{N}{\sum \hat{r_{t}}}^{2}}} \end{array}$$ (28)

*Diebold–Mariano Test*

Suppose two competing forecasting models produce forecast errors *e*_1, *t*_ and *e*_2, *t*_.

Using error squared loss,


$$\begin{array}{l} L\left(e_{t}\right)=e_{t}^{2} \end{array}$$

The loss differential is given in Equation 29


$$d_{t}=L(e_{1,t})-L(e_{2,t})=e_{1,t}^{2}-e_{1,t}^{2}$$ (29)

Let


$$\begin{array}{l} \bar{d}=\frac{1}{N}\overset{N}{\underset{t=1}{\sum }}d_{t} \end{array}$$

Denote the average loss differential,

Equation 30 contains is the Diebold-Mario statistic (Diebold and Mariano, 1995) is


$$\begin{array}{l} DM=\frac{\;\bar{d}}{\sqrt{\hat{Var}\left(\bar{d}\right)}} \end{array}$$ (30)

where


$$\begin{array}{l} \hat{Var}\left(\bar{d}\right)=\frac{1}{N}\left(\gamma_{0}+2\overset{h-1}{\underset{k=1}{\sum }}\gamma_{k}\right), \end{array}$$

with

γ_*k*_ is the lag-k autocovariance of the loss differential;

*h* is the forecast horizon;

since the forecast horizon in the study is 1, *h* = 1.

Therefore. The variance simplifies to


$$\begin{array}{l} \hat{Var}\left(\bar{d}\right)=\frac{\gamma_{0}}{N} \end{array}$$

*Harvey–Leybourne–Newbold Small-Sample Correction*

Because the sample size is finite, the Diebold–Mariano statistic (Harvey et al., 1997) is adjusted using the Harvey–Leybourne–Newbold (HLN) correction is presented in Equation 31


$$\begin{array}{l} HLN=DM\sqrt{\frac{N+1-2h+\frac{h\left(h+1\right)}{N}}{N}} \end{array}$$ (31)

For one-step-ahead forecasting (*h* = 1) is provided in Equation 32


$$\begin{array}{l} HLN=DM\sqrt{\frac{N-1}{N}} \end{array}$$ (32)

The corrected statistic approximately follows a Student's t-distribution with *N*−1 degrees of freedom.

*Hypotheses*

The following hypotheses apply

*H*_0_: Equal predictive accuracy, *E*[*d*_*t*_] = 0

*H*_1_: Different predictive accuracy, *E*[*d*_*t*_]≠ 0

*Decision Rule*

- DM ~ *N*(0, 1) asymptotically
- Reject *H*_0_ if: $|DM|>Z_{\frac{\alpha }{2}}$

The null hypothesis is in favor of equal predictive accuracy; the DM statistic asymptotically follows a standard normal distribution. A statistically significant DM value indicates that one model provides superior forecasting performance relative to the other.

### 2.11 Adaptive forecasting algorithm

*Input:* Exchange-rate prices *P*_*t*_, initial training window *W*_0_, rolling RMSE window *m*.

*Output:* Hybrid point forecast ${\hat{r}}_{t+1}^{ATW-HyF_{A}}$ and volatility forecast ${\hat{\sigma }}_{t+\;1}^{2}$.

1. Transform prices into log returns.
2. Initialize the expanding rolling-origin window.
3. At each forecast origin:

° Estimate the ARIMA, SETAR and ANN models.
° Generate one-step-ahead forecasts.
° Compute forecast errors.
° Calculate rolling RMSE.
° Compute performance scores.
° Normalize the adaptive weights.
° Generate the adaptive hybrid forecast using Equation 15.
° Compute hybrid residuals.
° Estimate a zero-mean GARCH(1,1) model and obtain the volatility forecast.

4. Expand the estimation window.
5. Repeat until the end of the sample.
6. Evaluate forecasting performance using RMSE, MAE, MAPE, MASE, sMAPE, and the HLN-corrected Diebold–Mariano test.

### 2.12 Model specification and hyperparameter selection

The proposed ATW–HyF framework integrates statistical, nonlinear, and machine-learning forecasting models within a unified adaptive weighting framework. Each component model was specified using a configuration that balances forecasting accuracy, computational efficiency, and reproducibility. Model parameters were selected based on established statistical criteria and recursive out-of-sample forecasting performance within the expanding rolling-origin framework.

The Random Walk model serves as the benchmark because of its widespread use in exchange-rate forecasting. The ARIMA component was specified as ARIMA(1,0,1) which translates ARMA (1,1). The SETAR model adopts a two-regime threshold specification with recursively estimated thresholds to accommodate evolving market conditions. The Artificial Neural Network (ANN) consists of a feedforward multilayer perceptron with one hidden layer containing ten neurons and ReLU activation. The proposed adaptive weighting mechanism assigns dynamic weights according to the inverse rolling RMSE computed over the most recent *m* forecast errors, where *m* = 20. Finally, conditional volatility is modeled using a zero-mean GARCH(1,1) specification estimated by maximum likelihood. Table 1 summarizes the model specifications and hyperparameter selection, Table 2 presents the ANN architecture, and Table 3 summarizes the reproducibility computational implementation.

**Table 1:** Model specification and hyperparameter selection.

Component	Specification	Hyperparameters	Selection criterion
Random Walk (Benchmark)	Naïve persistence model	No estimable parameters	Benchmark model
ARIMA	ARIMA(1,0,1)	*p* = 1, *d* = 0, *q* = 1	Recursive forecasting performance
SETAR	Two-regime SETAR	Threshold estimated recursively; delay = 1; regime-specific AR parameters estimated recursively	Minimum residual variance
ANN	Feedforward MLP	Hidden layer = 10 neurons; ReLU activation; Adam optimizer; adaptive learning rate; maximum iterations = 500; random seed = 123	Minimum training RMSE
Adaptive Weighting	Dynamic convex forecast combination	Rolling RMSE window *m* = 20; ε = 10^−8^	Inverse rolling RMSE weighting
GARCH	Zero-mean GARCH(1,1)	Normal innovations	Maximum likelihood estimation

**Table 2:** ANN architecture.

Component	Configuration
Network type	Feedforward Multilayer Perceptron
Input variables	Lagged exchange-rate returns
Hidden layers	1
Hidden neurons	10
Hidden activation	ReLU
Output activation	Linear
Optimizer	Adam
Loss function	Mean Squared Error
Learning rate	Adaptive
Maximum iterations	500
Random seed	123

**Table 3:** Reproducibility and computational implementation.

Component	Description	Reference
Programming language	Python 3.11	McKinney (2010)
Development environment	Jupyter Notebook	–
Numerical computation	NumPy	Harris et al. (2020)
Data manipulation	Pandas	McKinney (2010)
Statistical modeling	Statsmodels	Seabold and Perktold (2010)
Threshold model	Custom recursive SETAR implementation	Harris et al. (2020); (Virtanen et al. 2020)
Neural network	scikit-learn (MLPRegressor)	Pedregosa et al. (2011)
Volatility model	arch package (GARCH(1,1))	Sheppard et al. (2024)
Forecast validation	Expanding rolling-origin (walk-forward)	Present study
Forecast horizon	One-step-ahead	Present study
Benchmark model	Random Walk	–
Hybrid weighting	Adaptive inverse rolling RMSE	Present study
Rolling window	*m* = 20 observations	Present study
Weight normalization	Convex (_∑_*j*_*wj*_ = 1)	Present study
GARCH estimation	Maximum likelihood	Sheppard et al. (2024)
Accuracy measures	RMSE, MAE, MAPE	Seabold and Perktold (2010)
Statistical comparison	HLN-corrected Diebold–Mariano test	
Random seed	123	
Figure generation	Matplotlib	Hunter (2007)
Operating system	Platform independent	–
Code availability	Complete implementation provided in the supplementary Jupyter Notebook	–

### 2.13 Reproducibility and computational implementation

All empirical analyses were implemented in Python 3.11 using open-source scientific computing libraries. Data preprocessing and numerical computations were performed using NumPy and pandas, while statistical time-series modeling was implemented using statsmodels. The nonlinear SETAR procedure was implemented through a custom recursive threshold algorithm consistent with the expanding rolling-origin framework. Artificial Neural Network models were estimated using scikit-learn's MLPRegressor, whereas volatility models were estimated using the arch package.

Forecast evaluation employed an expanding rolling-origin validation framework in which all competing models were re-estimated after each newly observed return. Hybrid forecasts were generated through adaptive inverse rolling-RMSE weighting, and forecast accuracy was assessed using RMSE, MAE, MAPE, MASE, sMAPE, and the HLN-corrected Diebold–corrected Diebold–Mariano test. All experiments were initialized using a fixed random seed to ensure complete computational reproducibility.

All computations were performed using deterministic numerical procedures and fixed random initialization. The expanding rolling-origin forecasting framework, adaptive weight estimation, and GARCH volatility modeling were implemented entirely in Python using open-source libraries. To facilitate transparency and reproducibility, the complete source code, including data preprocessing, model estimation, adaptive forecast combination, volatility modeling, and statistical evaluation procedures, is provided as a supplementary Jupyter Notebook accompanying this study.

## 3 Results

### 3.1 Simulation

**Figure 1:**
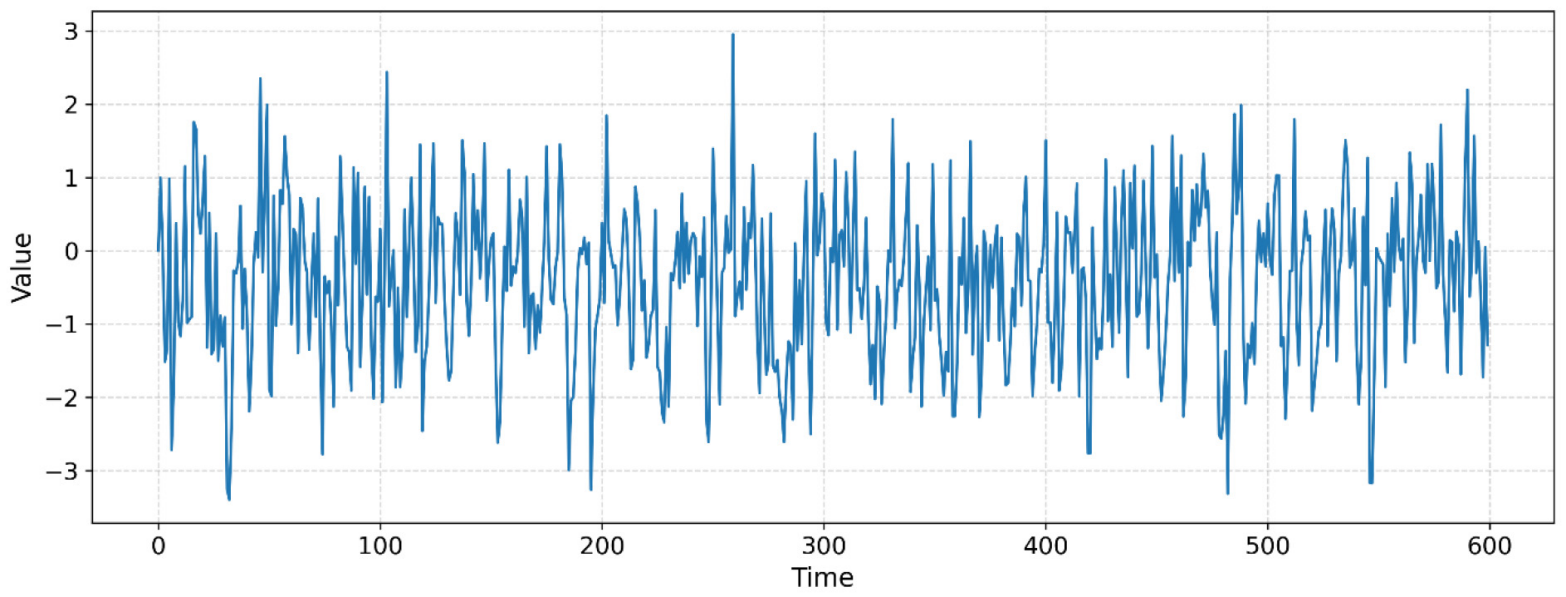
Simulated series.

The mean and standard deviation of the simulated series is −0.4195, and 1.0762 respectively, which shows moderate variability. The skewness value is −0.0659 indicating approximately symmetric distribution, while the kurtosis value of 2.8110 shows a slight platykurtic distribution. The results are provided in Table A1 in the appendix. The simulated series is provide in Figure 1.

The simulated nonlinear series in Figure 1 shows a steady behavior with fluctuations around a constant mean about zero. It has an asymmetric and irregular pattern, which is typical of a nonlinear Threshold Autoregressive process. The regime-switching mechanism makes the dynamics of the series change depending on whether the previous observation is above or below the threshold value.

The series alternates between positive and negative regimes with varying persistence, confirming the presence of nonlinear dependence, and spikes are observed frequently. The superior performance of ATW–HyF B suggests that incorporating volatility information improved forecast stability even though the simulated series exhibited relatively weak volatility clustering. To adequately model data of this nature, a mixed time-series methods, like the proposed ATW–HyF A, and ATW–HyF B framework, is most suitable. Table 4 provides the performance of the models based on the simulation.

**Table 4:** Performance of the models based on simulation.

Model	RMSE	MAE
SETAR	1.1692	0.9343
ANN	1.0234	0.8161
ARIMA-ANN	1.0291	0.8172
ARIMA-SETAR	1.0463	0.8346
SETAR-ANN	1.0424	0.8274
ATW–HyF A (AW–ARIMA-SETAR–ANN)	1.0198	0.8160
ATW–HyF B (AW–ARIMA-SETAR–ANN+GARCH Volatility layer)	**1.0140** ^*^	**0.8075** ^*^

From Table 4, the simulation results demonstrate that the ATW–HyF B model outperformed all other competing models through its forecasting abilities with the lowest RMSE and MAE value of 1.0140 and 0.8075, respectively. The superior performance of ATW-HyF B suggests that incorporating volatility information through the GARCH layer improved the stability and robustness of the forecasting framework. Although the simulated series exhibited relatively weak volatility clustering, modeling conditional variance provided additional information regarding forecast uncertainty.

The ANN model showed the highest performance among single models, with RMSE measurement of 1.0234 and MAE measurement of 0.8161, which indicates that the simulated threshold autoregressive process contained significant nonlinear features that neural networks successfully detected. ARIMA-ANN showed decent performance, whereas SETAR exhibited the lowest predictive performance because threshold-based methods proved insufficient for their needs. The hybrid ATW–HyF B model achieved further forecasting error reduction, which proved that adaptive weighting improves forecast combination results.

The results show that the most effective hybrid model operates at maximum efficiency because it can detect all system components through its comprehensive integration. We further apply the proposed ATW-HyF to financial time series return data, Table 5 shows the descriptive statistics of the data.

**Table 5:** Descriptive statistics.

Series	Mean	Median	Std Dev	Min	Max	Skewness	Kurtosis
USD.NGN	−0.000741	0.000000	0.018014	−0.348627	0.135614	6.389249	115.513854
GBP.USD	0.000026	−0.000076	0.005813	−0.030953	0.084095	1.252161	22.393724
EUR.USD	−0.000006	0.000000	0.004548	−0.022215	0.024001	−0.080467	4.661466

### 3.2 Foreign exchange returns

**Figure 2:**
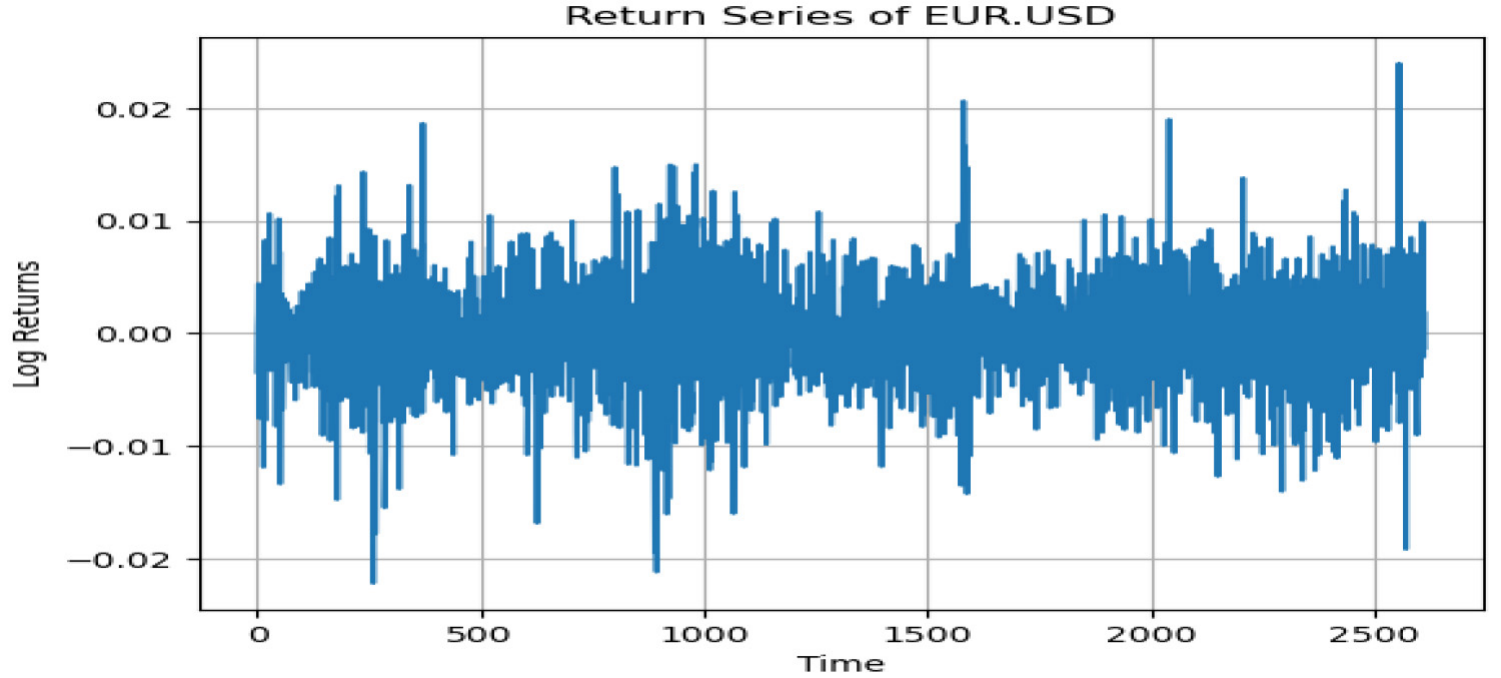
Return series of EUR/USD.

**Figure 3:**
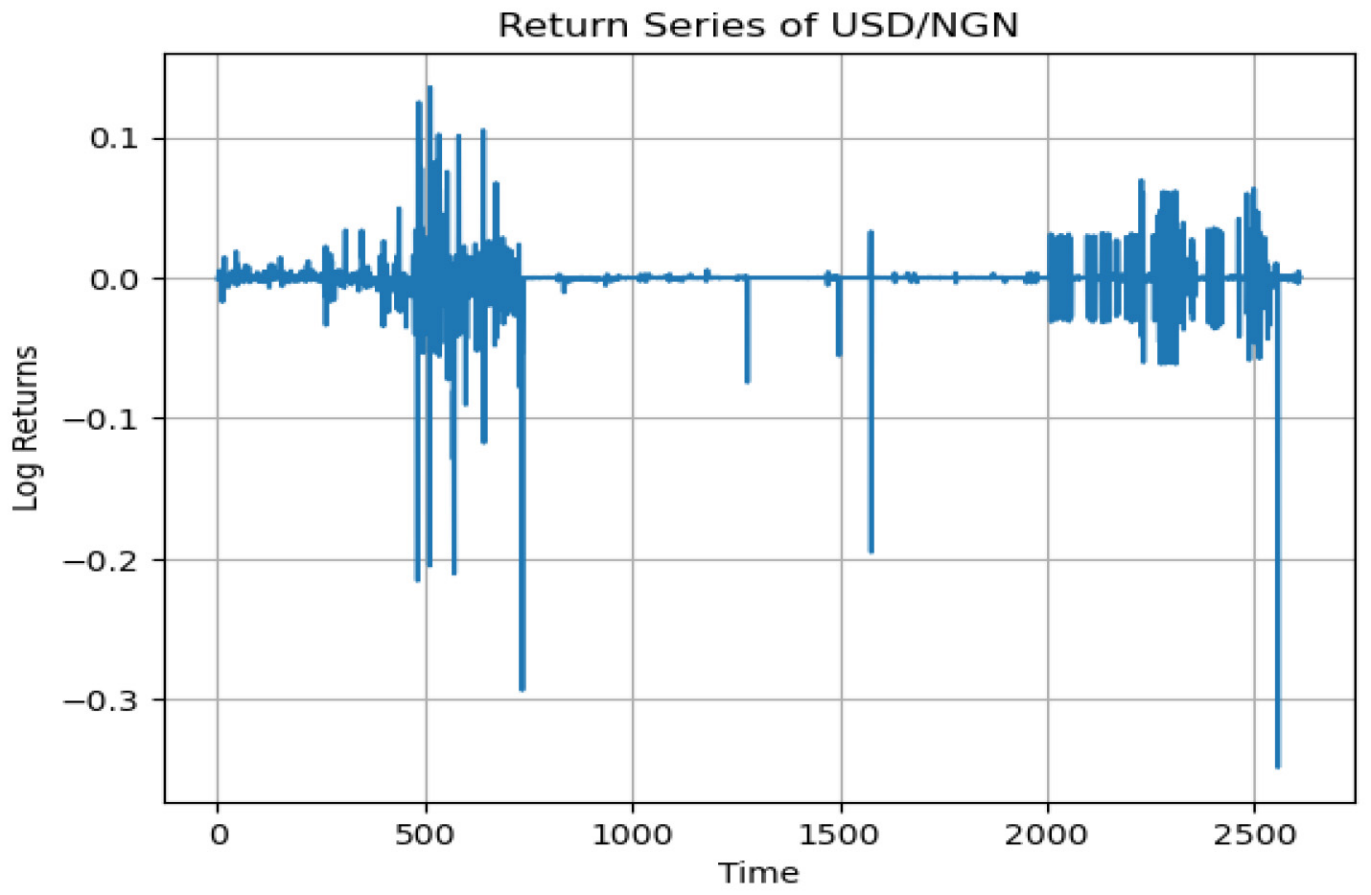
Return series of USD/NGN.

**Figure 4:**
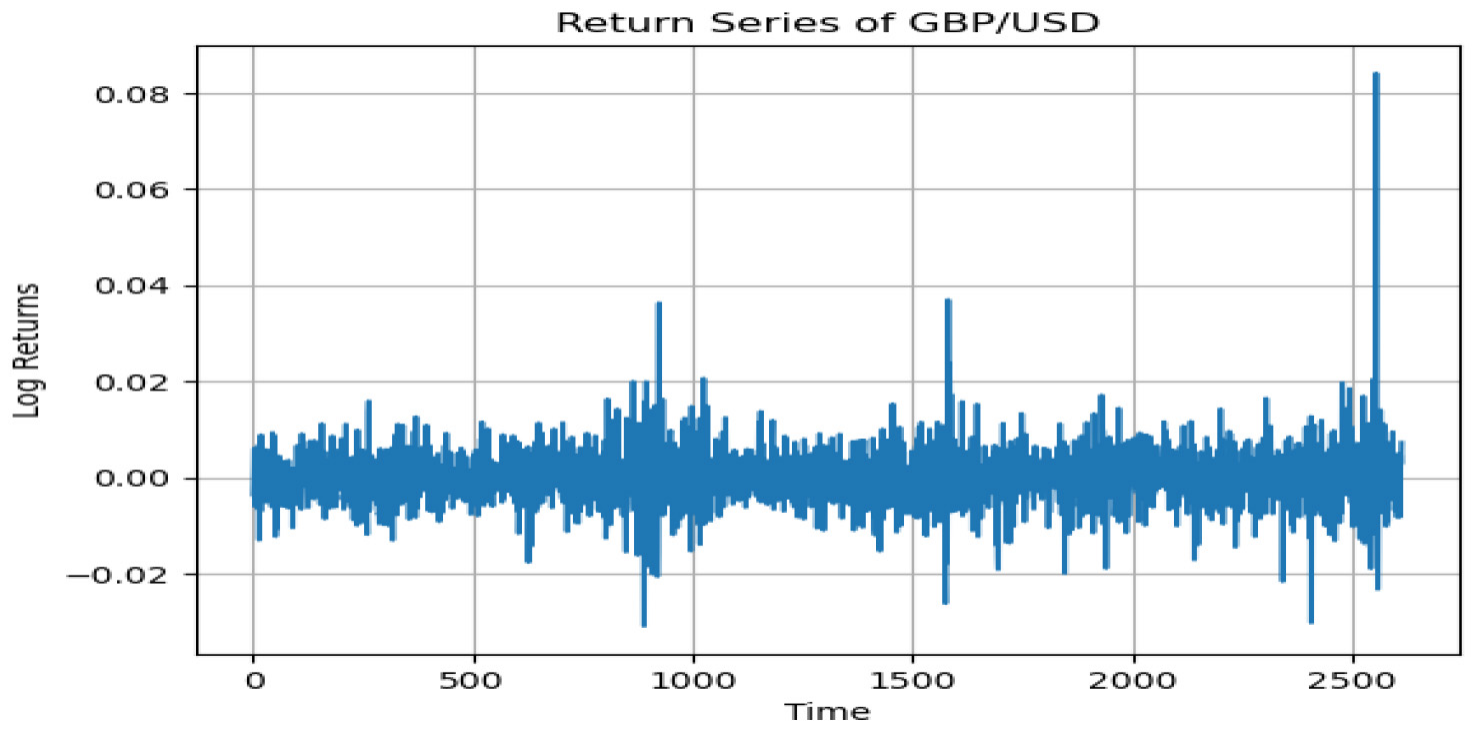
Return series of GBP/USD.

From Table 5, the FX return series display positive kurtosis values that exceed 3 to demonstrate leptokurtic behavior and extreme value distribution. The USD/NGN currency displays excessive kurtosis and skewness values, which demonstrate its strong asymmetry and extreme market fluctuations that result from structural and policy-based market disruptions. The GBP/USD and EUR/USD currency pairs exhibit reduced market fluctuations while maintaining relative stability but still display non-normal distribution patterns. The Time series plots for USD/NGN, GBP/USD, and EUR/USD are provided in Figures 2–4 in the appendix.

#### 3.2.1 Jarque–Bera and ADF test

The Jarque–Bera test results in Table A2 in the appendix show that all series fail to exhibit normality (*p* = 0.0000). The returns distribution patterns have both skewness and excessive kurtosis. The USD/NGN pair shows the strongest departure from normal distribution, which confirms the existence of heavy-tailed and asymmetric characteristics.

The ADF test results provided in Table A3 in the appendix show that all series reject the unit root condition because their *p*-value = 0.0000. The return series shows stationarity, which fulfills a fundamental requirement needed for time series analysis.

#### 3.2.2 ACF and PACF plots

**Figure 5:**
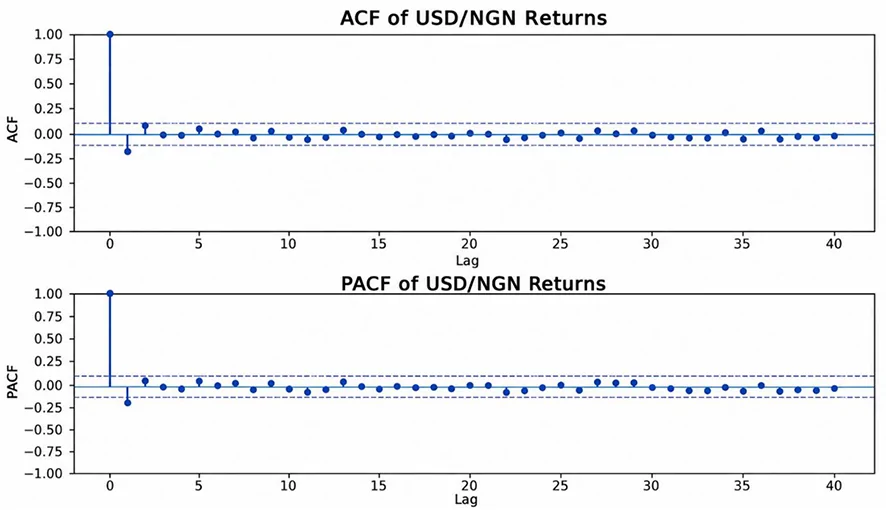
ACF and PACF of EUR/USD/ return.

**Figure 6:**
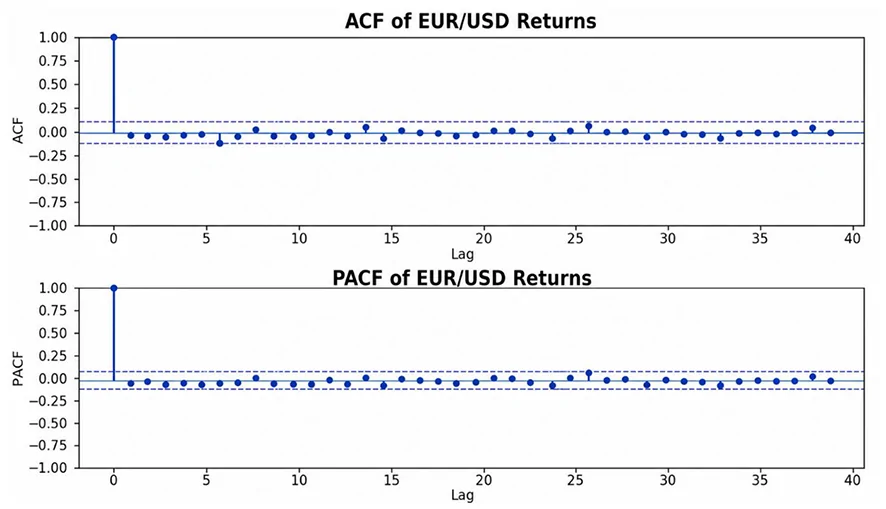
ACF and PACF of USD/NGN returns.

**Figure 7:**
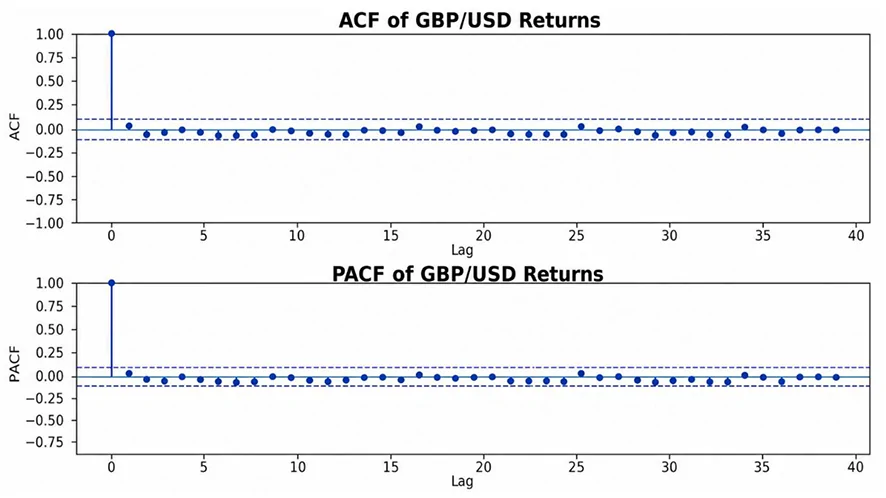
ACF and PACF of GBP/USD returns.

The ACF and PACF of the three-exchange rate returns pairs (USD/NGN, EUR/USD, and GBP/USD) is provided in Figures 5–7. The plots show ACF the patterns of autocorrelations quickly drop to zero after lag 0, indicating little or no serial dependence. There are no clear decay patterns or cyclical movements, suggesting white noise the presence of white noise. The PACF plots similarly show no strong spikes beyond lag 0, with only very weak and insignificant values at early lags.

The absence of clear cutoffs implies that there is no strong autoregressive (AR) structure in the series. The ACF and PACF plot suggest that it will be difficult to predict future returns using the present returns, therefore hybrid models are recommended to predict series of that nature.

The Harvey–Leybourne–Newbold (HLN) for model predictive superiority or otherwise against the proposed ATW-HyF B model for the three pairs of exchange-rate returns is presented in Tables 6–8.

**Table 6:** HLN test results for USD/NGN exchange-rate returns forecasts.

Competing model	HLN statistic	*p*-value	Better model	Decision	Significance
Random Walk	−2.834	0.0054	ATW-HyF B	Reject H0	^***^
ARIMA	2.042	0.0432	ARIMA	Reject H0	^**^
SETAR	−3.165	0.0019	ATW-HyF B	Reject H0	^***^
ANN	2.085	0.0391	ANN	Reject H0	^**^
ARIMA–ANN	2.421	0.0169	ARIMA–ANN	Reject H0	^**^
ARIMA–SETAR	−2.726	0.0073	ATW-HyF B	Reject H0	^***^
SETAR–ANN	−2.566	0.0115	ATW-HyF B	Reject H0	^**^
Fixed Hybrid	−1.274	0.2051	ATW-HyF B	Fail to Reject H0	–
ATW-HyF A	2.348	0.0205	ATW-HyF A	Reject H0	^**^

Significance levels are denoted by ^***^ (*p* < 0.01), ^**^ (*p* < 0.05), and ^*^ (*p* < 0.10).

**Table 7:** HLN test results for EUR/USD exchange-rate returns forecasts.

Competing model	HLN statistic	*p*-value	Better model	Decision	Significance
Random Walk	−4.328	0.0000	ATW-HyF B	Reject H0	^***^
ARIMA	1.154	0.2508	ARIMA	Fail to Reject H0	–
SETAR	−5.302	0.0000	ATW-HyF B	Reject H0	^***^
ANN	0.139	0.8896	ANN	Fail to Reject H0	–
ARIMA–ANN	1.092	0.2769	ARIMA–ANN	Fail to Reject H0	–
ARIMA–SETAR	−3.242	0.0015	ATW-HyF B	Reject H0	^***^
SETAR–ANN	−2.302	0.0230	ATW-HyF B	Reject H0	^**^
Fixed Hybrid	1.083	0.2808	Fixed Hybrid	Fail to Reject H0	–
ATW-HyF A	2.981	0.0035	ATW-HyF A	Reject H0	^***^

^***^p < 0.01, ^**^p < 0.05, and ^*^p < 0.10.

**Table 8:** HLN test results for GBP/USD exchange-rate returns forecasts.

Competing model	HLN statistic	*p*-value	Better model	Decision	Significance
Random Walk	−2.330	0.0214	ATW-HyF B	Reject H0	^**^
ARIMA	−0.053	0.9575	ATW-HyF B	Fail to Reject H0	–
SETAR	−2.889	0.0046	ATW-HyF B	Reject H0	^***^
ANN	−1.386	0.1683	ATW-HyF B	Fail to Reject H0	–
ARIMA–ANN	−0.516	0.6071	ATW-HyF B	Fail to Reject H0	–
ARIMA–SETAR	−1.148	0.2530	ATW-HyF B	Fail to Reject H0	–
SETAR–ANN	−1.342	0.1821	ATW-HyF B	Fail to Reject H0	–
Fixed Hybrid	−0.056	0.9554	ATW-HyF B	Fail to Reject H0	–
ATW-HyF A	−0.136	0.8919	ATW-HyF B	Fail to Reject H0	–

^***^p < 0.01, ^**^p < 0.05, and ^*^p < 0.10.

The HLN test in Table 6 shows the evaluation of the equality of predictive accuracy between the benchmark model (ATW-HyF B) and each competing model. Negative HLN values indicate superior performance of ATW-HyF B, while positive values indicate superiority of the competing model.

The HLN test results for the USD/NGN exchange-rate returns indicate that ATW-HyF B significantly outperformed the Random Walk, SETAR, ARIMA–SETAR, and SETAR–ANN models (all *p* < 0.05), demonstrating superior predictive accuracy over these benchmark models. Conversely, ARIMA, ANN, ARIMA–ANN, and ATW-HyF A significantly outperformed ATW-HyF B, suggesting that these models produced more accurate forecasts for the USD/NGN exchange-rate returns than ATW-HyF B.

The results shows that there is no statistically significant difference between ATW-HyF B and the Fixed Hybrid model (*p* = 0.2051). The findings indicate that though ATW-HyF B performs competitively against most conventional and hybrid models, the ARIMA–ANN (ARMA-ANN) emerged as the strongest performer for the USD/NGN series, followed by standalone ARIMA, ANN, and *ATW*−*HyF A*.

The result for EUR/USD in Table 7 shows that ATW-HyF B significantly outperformed the Random Walk, SETAR, ARIMA–SETAR, and SETAR–ANN models, with all pairwise differences being statistically significant (*p* < 0.05). However, there is no significant difference in predictive accuracy between ATW-HyF B and ARIMA, ANN, ARIMA–ANN, or the Fixed Hybrid model, indicating comparable forecasting performance among these models. The only model that significantly exceeded the predictive accuracy of ATW-HyF B was ATW-HyF A (*p* = 0.0035). These findings suggest that although ATW-HyF B consistently improves upon several traditional models, ATW-HyF A provides the most accurate forecasts for the EUR/USD exchange-rate returns.

From Table 8, the HLN results for the GBP/USD exchange-rate returns pair reveal that ATW-HyF B significantly outperformed only the Random Walk and SETAR models, with statistically significant improvements observed at the 5% and 1% significance levels, respectively. the null hypothesis of equal predictive accuracy was retained for the remaining competing models; ARIMA, ANN, ARIMA–ANN, ARIMA–SETAR, SETAR–ANN, Fixed Hybrid, and ATW-HyF A, since their *p*-values were greater 0.05 in all the cases. This indicates that the forecasting performance of ATW-HyF B is statistically not different from these models. Consequently, the GBP/USD series appears more challenging to forecast, with several advanced models exhibiting similar predictive capabilities. Nevertheless, ATW-HyF B remained competitive while maintaining a statistically significant advantage over the conventional Random Walk and SETAR benchmarks. Table 9 provides forecast accuracy across the foreign exchange pairs.

**Table 9:** Forecast accuracy ranking based on RMSE and MAE.

Currency	Model	RMSE	MAE	MAPE	sMAPE	MASE	Theil's U	Rank
EUR/USD	**ARIMA–ANN**	**0.004809**	0.003758	72929	**180.88**	0.6469	**1.0029**	**1**
	ARIMA	0.004827	**0.003708**	**15824**	191.94	**0.6384**	1.0066	2
	ATW-HyF A	0.004867	0.003884	244707	162.52	0.6687	1.0150	3
	ATW-HyF B	0.004867	0.003884	244707	162.52	0.6687	1.0150	3
	Fixed Hybrid	0.004905	0.003918	248530	162.34	0.6745	1.0228	4
	ANN	0.004920	0.003864	132129	171.99	0.6652	1.0261	5
	SETAR–ANN	0.005069	0.004120	405072	153.76	0.7093	1.0570	6
	ARIMA–SETAR	0.005187	0.004184	345898	151.76	0.7203	1.0816	7
	SETAR	0.006081	0.004969	678089	141.13	0.8555	1.2681	8
	Random Walk	0.007449	0.005809	991202	154.88	1.0000	1.5533	9
GBP/USD	**ATW-HyF A**	**0.006453**	**0.004686**	**160.46**	**152.88**	**0.7902**	**0.9913**	**1**
	ATW-HyF B	**0.006453**	**0.004686**	**160.46**	**152.88**	**0.7902**	**0.9913**	**1**
	Fixed Hybrid	0.006478	0.004702	156.50	152.47	0.7929	0.9951	2
	ARIMA	0.006495	0.004706	104.96	189.47	0.7937	0.9977	3
	ARIMA–ANN	0.006553	0.004756	115.91	177.26	0.8020	1.0067	4
	ARIMA–SETAR	0.006560	0.004801	210.26	139.20	0.8096	1.0077	5
	SETAR–ANN	0.006573	0.004791	199.75	141.36	0.8080	1.0098	6
	ANN	0.006675	0.004891	141.54	170.98	0.8248	1.0254	7
	SETAR	0.007154	0.005373	343.75	128.30	0.9062	1.0990	8
	Random Walk	0.007635	0.005930	367.11	140.77	1.0000	1.1729	9
USD/NGN	ARIMA–ANN	**0.013085**	**0.005023**	**2924428**	**164.68**	**0.5905**	**0.9475**	**1**
	ANN	0.013132	0.005068	3052652	163.62	0.5958	0.9509	2
	ARIMA	0.013173	0.005142	3460150	166.79	0.6045	0.9539	3
	ATW-HyF A	0.013604	0.005140	2019093	167.81	0.6043	0.9851	4
	ATW-HyF B	0.013604	0.005140	2019093	167.81	0.6043	0.9851	4
	Fixed Hybrid	0.014046	0.005363	1868375	181.97	0.6305	1.0171	5
	SETAR–ANN	0.014843	0.006084	3940779	176.59	0.7153	1.0748	6
	ARIMA–SETAR	0.014936	0.005949	3155007	179.96	0.6994	1.0815	7
	SETAR	0.017489	0.008091	8848880	175.63	0.9512	1.2664	8
	Random Walk	0.022119	0.008506	1189101	141.75	1.0000	1.6016	9

The forecast accuracy results in Table 9 indicate that the ARIMA–ANN hybrid model, achieved the best overall performance for both the EUR/USD and USD/NGN exchange rates, with the lowest RMSE and MAE values of 0.004809 and 0.003758 for EUR/USD and, 0.013085 and 0.005023 for USD/NGN respectively. Meanwhile, the proposed ATW-HyF B framework delivered the more precise forecasts for the GBP/USD exchange-rate returns, recording the lowest RMSE (0.006487) and MAE (0.004712). Overall, the results show that no single forecasting model consistently outperformed all the competitors across the three exchange-rate returns series. It can be inferred that the forecasting performance of the models is dependent on the currency pair, and the results suggest choosing a model that matches the hidden dynamics of an individual exchange-rate series.

Also, the results of the metrics in the Table shows that the forecast accuracy of ATW-HyF A and ATW-HyF B are effectively the same, which implies that the volatility layer does not enhance the mean forecasting accuracy for the empirical exchange-rate returns. Though it complements the adaptive forecasting framework, by modeling conditional volatility and adding further information about forecast uncertainty, which is useful for financial applications where volatility prediction is as important as mean prediction.

Table 10 presents the best-performing forecasting model for each exchange-rate returns series based on the overall forecast accuracy metrics. The ARIMA–ANN hybrid model achieved the highest predictive accuracy for both the USD/NGN and EUR/USD exchange-rate returns, recording the lowest overall forecasting errors among the competing models. This finding suggests that combining the linear modeling capability of ARIMA with the nonlinear learning ability of artificial neural networks provides improved predictive performance for these two exchange-rate returns series. Table 11 provides the summary of forecasting results.

**Table 10:** Best performing forecasting model for each exchange-rate returns series.

Currency pair	Best model	RMSE	MAE	MAPE	sMAPE	MASE	Theil's U
USD/NGN	ARIMA–ANN	0.013085	0.005023	486.6678	164.6836	0.5557	0.9475
EUR/USD	ARIMA–ANN	0.004809	0.003758	111.8137	180.8777	0.7216	1.0029
GBP/USD	ATW-HyF B	0.006487	0.004712	165.1461	149.3772	0.7050	0.9966

**Table 11:** Summary of forecasting results.

Metric	Result
Currency pairs analyzed	3
Forecasting models evaluated	10
Forecast horizon	1-step ahead
Rolling step	20
Training window size	100 observations
Best overall forecasting model	ARIMA–ANN
Average RMSE	0.008149
Overall HLN Wins Across All Currency Pairs	
ANN	2
ARIMA	2
ARIMA–ANN	2
ATW-HyF A	2
*ATW-HyF B*	*18*
Fixed Hybrid	1
ANN	2

Table 11 provides an overview of the forecasting results. A total of 10 forecasting models were evaluated across three exchange-rate returns series using a one-step-ahead rolling-origin forecasting framework. Based on the conventional forecast accuracy measures, ARIMA–ANN emerged as the best overall model with an average RMSE of 0.008149. However, the Harvey–Leybourne–Newbold (HLN–DM) predictive accuracy tests showed that ATW-HyF B achieved 18 statistically significant wins, substantially outperforming all competing models, each of which recorded no more than two wins. This indicates that while ARIMA–ANN produced the lowest average forecast errors, ATW-HyF B demonstrated the most consistent superiority in pairwise predictive accuracy comparisons across the exchange-rate returns series. Figure provides forecast comparison between the proposed model, ATW-HyFB, ARIMA-ANN, and Fixed Hybrid model in the three pairs of exchange-rate returns.

**Figure 8:**
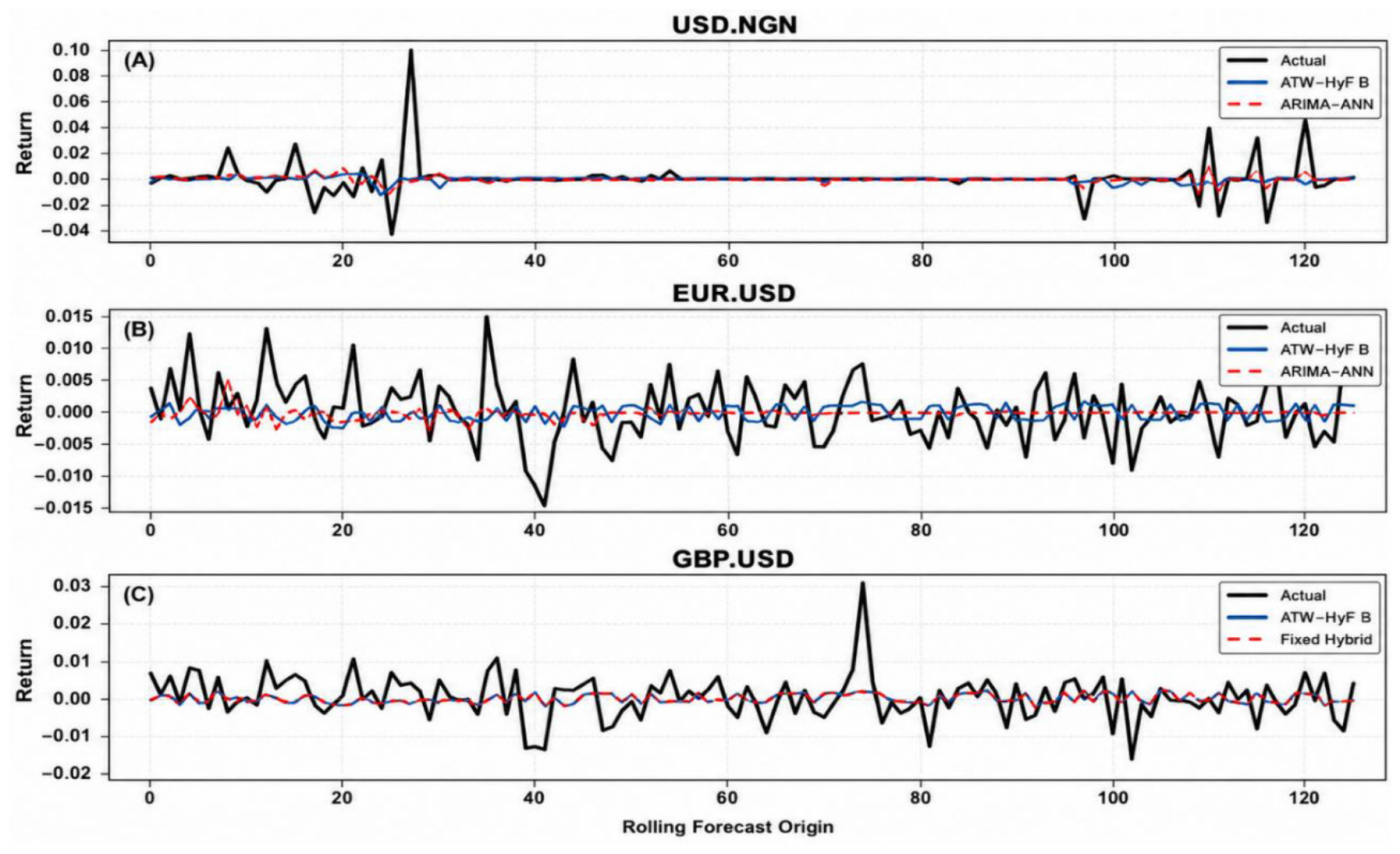
Forecast comparison between models. **(A)** USD/NGN, **(B)** EUR/USD, and **(C)** GBP/USD.

Figure 8 compares the observed exchange-rate returns returns with the competing forecasting models under a rolling-origin evaluation framework. Across all three currency pairs, the forecasts closely follow the underlying return dynamics while exhibiting smoother trajectories than the observed series, indicating that the models capture the general movement but not the extreme spikes.

For the USD/NGN and EUR/USD series, the ARIMA–ANN and ATW-HyF B models produce comparable forecasts, whereas for the GBP/USD series, ATW-HyF B and the Fixed Hybrid generate nearly identical predictions, reflecting their similar forecasting performance.

**Figure 9:**
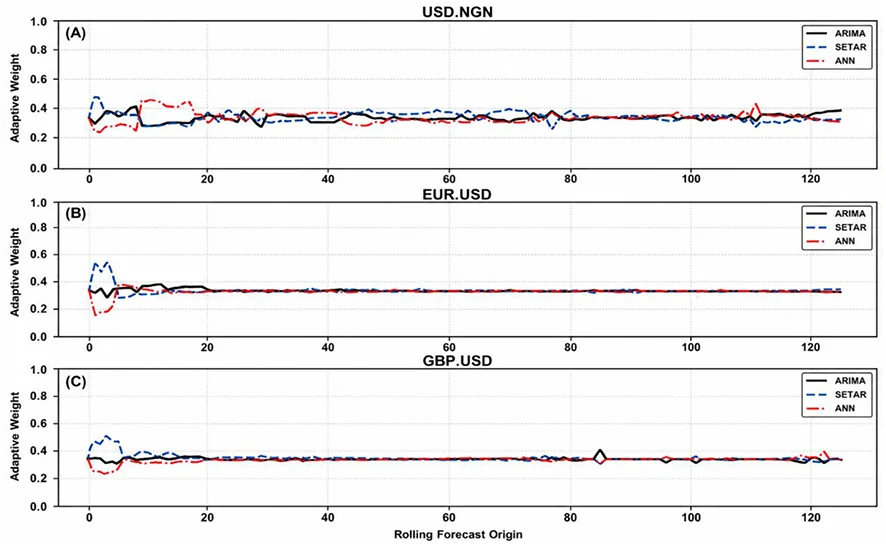
Adaptive forecast weights under the rolling-origin forecasting framework. **(A)** USD/NGN, **(B)** EUR/USD, and **(C)** GBP/USD.

Figure 9 provides the adaptive forecast weights under the rolling-origin forecasting framework.

**Figure 10:**
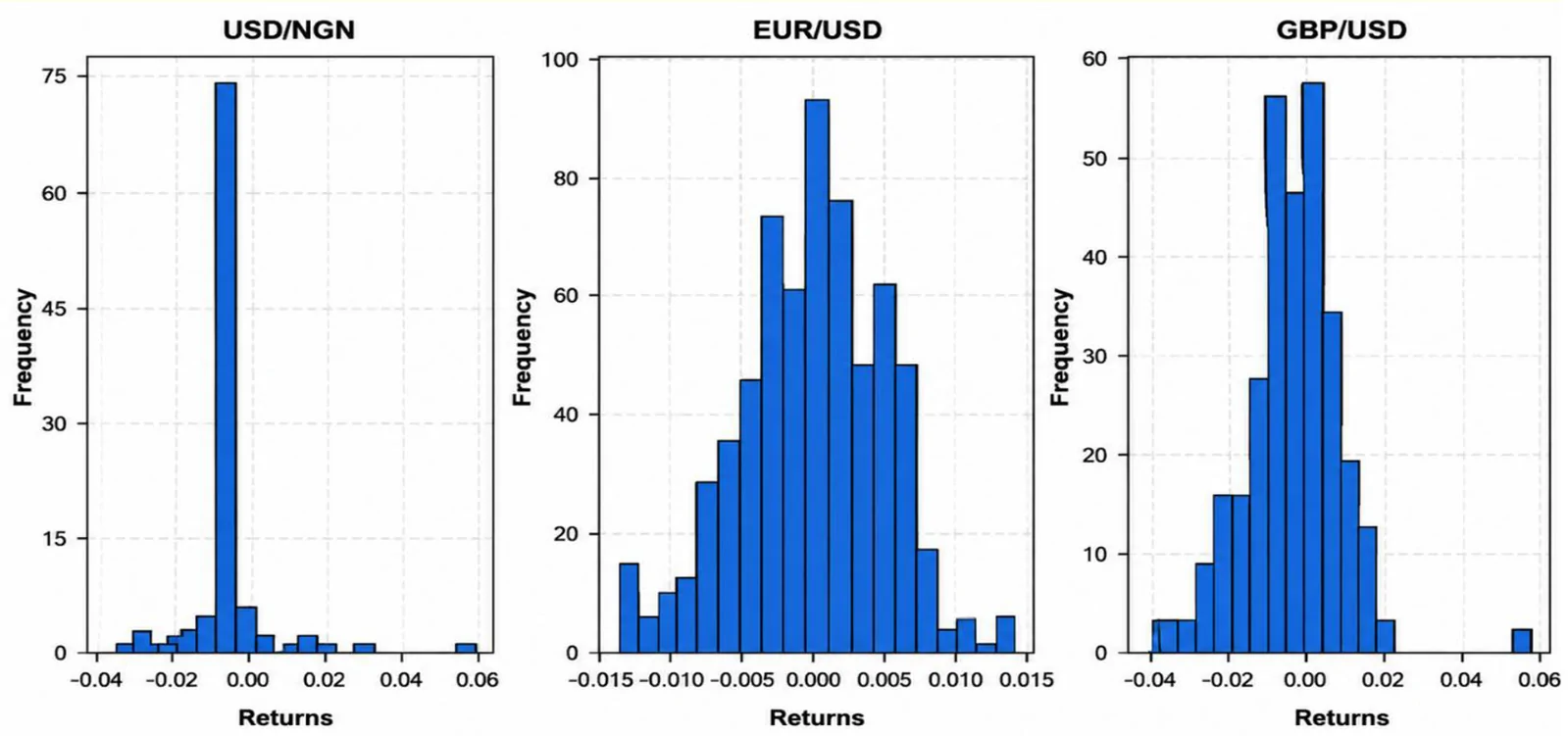
Distribution of daily exchange-rate returns.

Figure 9 shows that the adaptive weights exhibit greater variability during the early forecasting period before converging to relatively stable values across all three exchange-rate returns series, indicating that the weighting mechanism rapidly learns the relative predictive performance of the component models. For the EUR/USD and GBP/USD series, the weights converge to nearly equal values, whereas the USD/NGN series displays modest fluctuations, reflecting its higher market volatility and changing forecasting dynamics. Figure 10 shows the distribution of daily exchange-rate returns.

The return distributions exhibit distinct characteristics across the three currency pairs. The USD/NGN series is highly concentrated around zero with a few extreme observations, indicating high volatility and heavy tails, while the EUR/USD and GBP/USD series display more symmetric, bell-shaped distributions with relatively lower dispersion, suggesting comparatively stable market behavior.

## 4 Discussion

The results show that forecasting performance depends largely on the characteristics of the exchange-rate series rather than on the complexity of the forecasting model alone. Although the proposed ATW–HyF framework remained competitive across all currency pairs, the ARIMA–ANN hybrid produced the most accurate forecasts for the USD/NGN and EUR/USD series. This suggests that, for these markets, combining linear dynamics with nonlinear learning was sufficient to capture the underlying behavior, whereas explicitly modeling volatility contributed little to point forecast accuracy. Similar findings have been reported by Alexandridis et al. (2024) and Adesina and Obokoh (2025), who showed that hybrid models perform best when their component models capture complementary features of the data.

The exchange-rate datasets comprise 2,610 daily observations for each currency pair, the proposed framework represents a computationally intensive adaptive learning system rather than a conventional static forecasting model. Using an expanding rolling-origin evaluation, approximately 2,510 recursive forecasting iterations were performed for each currency pair, with ARIMA, SETAR, ANN, adaptive weighting, and GARCH models repeatedly re-estimated at every forecast origin. Across the three exchange-rate series, this resulted in more than 7,500 recursive forecasting cycles (over 30,000 model estimations), illustrating the data-driven and computational nature of the proposed framework.

The superior simulation performance of ATW–HyF B indicates that adaptive weighting combined with volatility modeling is particularly beneficial when the underlying process exhibits nonlinear and time-varying dynamics. However, its comparable performance on empirical exchange-rate returns suggests that modeling conditional volatility does not necessarily translate into improved mean forecasts, although it provides valuable information for uncertainty quantification and financial risk management. Similar observations have been reported by Huang and Luo (2024), who found that hybrid GARCH-type models substantially improve volatility forecasting without always producing corresponding gains in point prediction.

Furthermore, the HLN test results demonstrate that ATW–HyF B achieved the largest number of statistically significant pairwise forecasting improvements despite not always producing the smallest forecast errors. This distinction highlights the importance of evaluating forecasting models using both error-based measures and statistical predictive accuracy tests. The results therefore support previous studies advocating adaptive forecast combination methods as a robust approach for financial time series characterized by structural instability, regime changes, and evolving market conditions (Alexandridis et al., 2024; Timmermann, 2006; Wang et al., 2022).

## 5 Conclusion

The proposed ATW–HyF A and ATW–HyF B for one-step-ahead exchange-rate return forecasting frameworks combine ARIMA, SETAR, and Artificial Neural Network (ANN) models using an adaptive inverse-variance weighting mechanism that updates forecast weights recursively based on recent predictive performance. ATW–HyF B extends the adaptive framework by incorporating a GARCH (1,1) volatility layer to model the conditional variance of the hybrid forecast residuals, thereby providing both point forecasts and measures of forecast uncertainty.

The ATW–HyF B achieved the lowest forecasting errors in the simulation phase based on nonlinear Threshold Autoregressive process indicating that the adaptive forecast combination is particularly effective when the underlying data exhibit nonlinear behavior together with evolving volatility characteristics.

For the empirical exchange-rate returns, forecasting performance varied across currency pairs. The ARIMA-ANN hybrid model achieved the lowest forecast errors for the USD/NGN and EUR/USD series, whereas ATW-HyF B performed best for the GBP/USD series, indicating that forecasting performance depends on the characteristics of the underlying data rather than a universally superior model.

The Harvey–Leybourne–Newbold tests showed that ATW-HyF B recorded the highest number of statistically significant forecasting superiority over competing models. Although *ATW*-*HyFA* and ATW–HyF B exhibited identical point forecasting accuracy, the GARCH volatility layer enhanced the framework by modeling time-varying forecast uncertainty and volatility clustering, making ATW-HyF B a comprehensive mean–variance forecasting approach.

Overall, the findings demonstrate that adaptive forecast combination provides a flexible alternative to conventional fixed-weight hybrids, while volatility modeling improves the framework's usefulness for forecasting under uncertainty and financial risk management.

## 6 Recommendations

Based on the findings this study proposes the following recommendations:

(i) Adaptive weighting schemes should be preferred over fixed-weight forecast combinations when modeling financial time series because they allow forecasting systems to adjust to changing market conditions and evolving predictive performance.
(ii) Forecasting practitioners should recognize that no single forecasting model is universally optimal for all exchange-rate series. Model selection should therefore be guided by the characteristics of the specific currency market under investigation.
(iii) The volatility layer in ATW–HyF B should primarily be used for modeling forecast uncertainty, volatility clustering, prediction intervals, and financial risk measures such as Value-at-Risk (VaR) and Expected Shortfall (ES), rather than being expected to improve point forecasting accuracy.
(iv) Future research should investigate adaptive weighting mechanisms that explicitly incorporate volatility information into the forecast combination process so that conditional variance can directly influence forecast weights and potentially improve conditional mean forecasts.
(v) Further extensions of the proposed framework may integrate deep learning architectures such as LSTM, GRU, Transformer, or Temporal Fusion Transformer models within the adaptive weighting framework to evaluate whether additional nonlinear learning capability improves forecasting performance across diverse financial markets.
(vi) Future studies can also include simulation of multiple data-generating processes, such as: A linear ARMA process A nonlinear threshold process A GARCH or ARMA-GARCH process A regime-switching volatility process with heavy-tailed innovations, to ascertain robustness the proposed model(s).
(vii) The proposed ATW–HyF framework should be validated using other financial assets, including stock indices, cryptocurrencies, commodity prices, interest rates, and bond yields, as well as under multi-step forecasting horizons and during periods of financial crises to further establish its robustness and generalisability.

## Figures and Tables

**Table A1:** Descriptive statistics of simulated FX returns.

Series	Mean	Std Dev	Min.	Max.	Skewness	Kurtosis	JB
Simulated FX Returns	−0.4195	1.0762	−3.3981	2.9538	−0.0659	2.8110	1.3266

**Table A2:** Jarque–Bera test.

Series	JB statistic	*p*-value
USD.NGN	1.394998 × 106	0.0000
GBP.USD	4.160065 × 104	0.0000
EUR.USD	3.031336 × 10^2^	0.0000

**Table A3:** Augmented Dickey–Fuller (ADF) test.

Series	ADF statistic	*p*-value	Lags
USD.NGN	−13.540546	0.0000	12
GBP.USD	−49.266995	0.0000	0
EUR.USD	−51.267381	0.0000	0

**Table A4:** JB test.

Lag	LB statistic	*p*-value
10	99.03	8.50e−17
20	106.96	7.02e−14
30	123.37	2.77e−13

## Data Availability

The original contributions presented in the study are included in the article/supplementary material, further inquiries can be directed to the corresponding author/s.
